# Supplementary material for: Modeling glioblastoma heterogeneity as a dynamic network of cell states
Source: Mol Syst Biol. 2021 Sep 16;17(9):e10105. doi: 10.15252/msb.202010105 (PMC8444284; doi:10.15252/msb.202010105)
Supplement: Supplementary file 6 — Source Data for Figure 5 [file MSB-17-e10105-s004.zip › Figure5A_sourcedata/GSEA_3071/hallmarks_stateVI.GseaPreranked.1623742557031/HALLMARK_HEME_METABOLISM.html]

Details for gene set HALLMARK\_HEME\_METABOLISM[GSEA]

|  || Dataset | state63071 |
| Phenotype | NoPhenotypeAvailable |
| Upregulated in class | na\_neg |
| GeneSet | HALLMARK\_HEME\_METABOLISM |
| Enrichment Score (ES) | -0.23346071 |
| Normalized Enrichment Score (NES) | -1.0254287 |
| Nominal p-value | 0.42298287 |
| FDR q-value | 0.6727008 |
| FWER p-Value | 1.0 |
Table: GSEA Results Summary

  

Fig 1: Enrichment plot: HALLMARK\_HEME\_METABOLISM      
 Profile of the Running ES Score & Positions of GeneSet Members on the Rank Ordered List

  

| PROBE | GENE SYMBOL | GENE\_TITLE | RANK IN GENE LIST | RANK METRIC SCORE | RUNNING ES | CORE ENRICHMENT || 1 | ELL2 |  |  | 156 | 0.655 | -0.0119 | No |
| 2 | SMOX |  |  | 196 | 0.604 | 0.0063 | No |
| 3 | NR3C1 |  |  | 395 | 0.492 | -0.0251 | No |
| 4 | CTSB |  |  | 471 | 0.464 | -0.0237 | No |
| 5 | MAP2K3 |  |  | 550 | 0.440 | -0.0242 | No |
| 6 | UROD |  |  | 596 | 0.426 | -0.0162 | No |
| 7 | TSPAN5 |  |  | 608 | 0.423 | 0.0011 | No |
| 8 | RIOK3 |  |  | 819 | 0.373 | -0.0394 | No |
| 9 | BCAM |  |  | 844 | 0.369 | -0.0283 | No |
| 10 | NEK7 |  |  | 920 | 0.357 | -0.0320 | No |
| 11 | TOP1 |  |  | 975 | 0.349 | -0.0302 | No |
| 12 | MOSPD1 |  |  | 996 | 0.347 | -0.0191 | No |
| 13 | CAST |  |  | 1070 | 0.334 | -0.0234 | No |
| 14 | PSMD9 |  |  | 1075 | 0.333 | -0.0084 | No |
| 15 | PICALM |  |  | 1116 | 0.328 | -0.0038 | No |
| 16 | CDC27 |  |  | 1259 | 0.308 | -0.0285 | No |
| 17 | OSBP2 |  |  | 1299 | 0.303 | -0.0247 | No |
| 18 | HMBS |  |  | 1315 | 0.301 | -0.0144 | No |
| 19 | SLC25A38 |  |  | 1455 | 0.285 | -0.0394 | No |
| 20 | HDGF |  |  | 1502 | 0.279 | -0.0387 | No |
| 21 | SLC2A1 |  |  | 1606 | 0.268 | -0.0545 | No |
| 22 | SLC10A3 |  |  | 1708 | 0.260 | -0.0701 | No |
| 23 | KLF3 |  |  | 1927 | -0.268 | -0.1179 | No |
| 24 | ALDH6A1 |  |  | 2091 | -0.294 | -0.1491 | No |
| 25 | DCUN1D1 |  |  | 2248 | -0.323 | -0.1770 | No |
| 26 | NFE2L1 |  |  | 2452 | -0.367 | -0.2158 | Yes |
| 27 | RBM38 |  |  | 2461 | -0.369 | -0.2003 | Yes |
| 28 | AGPAT4 |  |  | 2489 | -0.376 | -0.1897 | Yes |
| 29 | ADD1 |  |  | 2529 | -0.384 | -0.1821 | Yes |
| 30 | RNF19A |  |  | 2566 | -0.391 | -0.1733 | Yes |
| 31 | NNT |  |  | 2583 | -0.396 | -0.1587 | Yes |
| 32 | TFDP2 |  |  | 2612 | -0.403 | -0.1471 | Yes |
| 33 | ARHGEF12 |  |  | 2731 | -0.434 | -0.1591 | Yes |
| 34 | RBM5 |  |  | 2865 | -0.483 | -0.1728 | Yes |
| 35 | CLCN3 |  |  | 2969 | -0.525 | -0.1763 | Yes |
| 36 | ADD2 |  |  | 2997 | -0.540 | -0.1578 | Yes |
| 37 | HAGH |  |  | 2999 | -0.541 | -0.1320 | Yes |
| 38 | MBOAT2 |  |  | 3012 | -0.546 | -0.1091 | Yes |
| 39 | TNRC6B |  |  | 3045 | -0.560 | -0.0910 | Yes |
| 40 | FOXO3 |  |  | 3053 | -0.564 | -0.0658 | Yes |
| 41 | LMO2 |  |  | 3110 | -0.592 | -0.0529 | Yes |
| 42 | GYPC |  |  | 3169 | -0.638 | -0.0383 | Yes |
| 43 | DAAM1 |  |  | 3300 | -0.758 | -0.0380 | Yes |
| 44 | BTG2 |  |  | 3377 | -0.857 | -0.0180 | Yes |
| 45 | PRDX2 |  |  | 3409 | -0.912 | 0.0173 | Yes |
| 46 | IGSF3 |  |  | 3429 | -0.959 | 0.0582 | Yes |
Table: GSEA details [plain text format]

  

Fig 2: HALLMARK\_HEME\_METABOLISM: Random ES distribution      
 Gene set null distribution of ES for **HALLMARK\_HEME\_METABOLISM**
